# ^1^H, ^15^N, ^13^C backbone and Cβ resonance assignments for UBQLN1 UBA and UBAA domains

**DOI:** 10.1007/s12104-023-10127-5

**Published:** 2023-04-06

**Authors:** Gwen R. Buel, Xiang Chen, Olumide Kayode, Anthony Cruz, Kylie J. Walters

**Affiliations:** Protein Processing Section, Center for Structural Biology, Center for Cancer Research, National Cancer Institute, National Institutes of Health, Frederick, MD 21702 USA

**Keywords:** UBQLN, Ubiquitin-proteasome pathway, Ubiquitin-associated domain, Ubiquitin-associated adjacent domain, Shuttle factor

## Abstract

UBQLN1 functions in autophagy and proteasome-mediated protein degradation. It contains an N-terminal ubiquitin-like domain (UBL), a C-terminal ubiquitin-associated domain (UBA), and a flexible central region which functions as a chaperone to prevent protein aggregation. Here, we report the ^1^H, ^15^N, and ^13^C resonance assignments for the backbone (^N^H, N, C’, Cα, and Hα) and sidechain Cβ atoms of the UBQLN1 UBA and an N-terminally adjacent segment called the UBA-adjacent domain (UBAA). We find a subset of the resonances corresponding to the UBAA to have concentration-dependent chemical shifts, likely due to self-association. We also find the backbone amide nitrogen of T572 to be shifted upfield relative to the average value for a threonine amide nitrogen, a phenomenon likely caused by T572 Hγ1 engagement in a hydrogen bond with adjacent backbone carbonyl atoms. The assignments described in this manuscript can be used to study the protein dynamics of the UBQLN1 UBA and UBAA as well as the interaction of these domains with other proteins.

## Biological Context

**Fig. 1:**
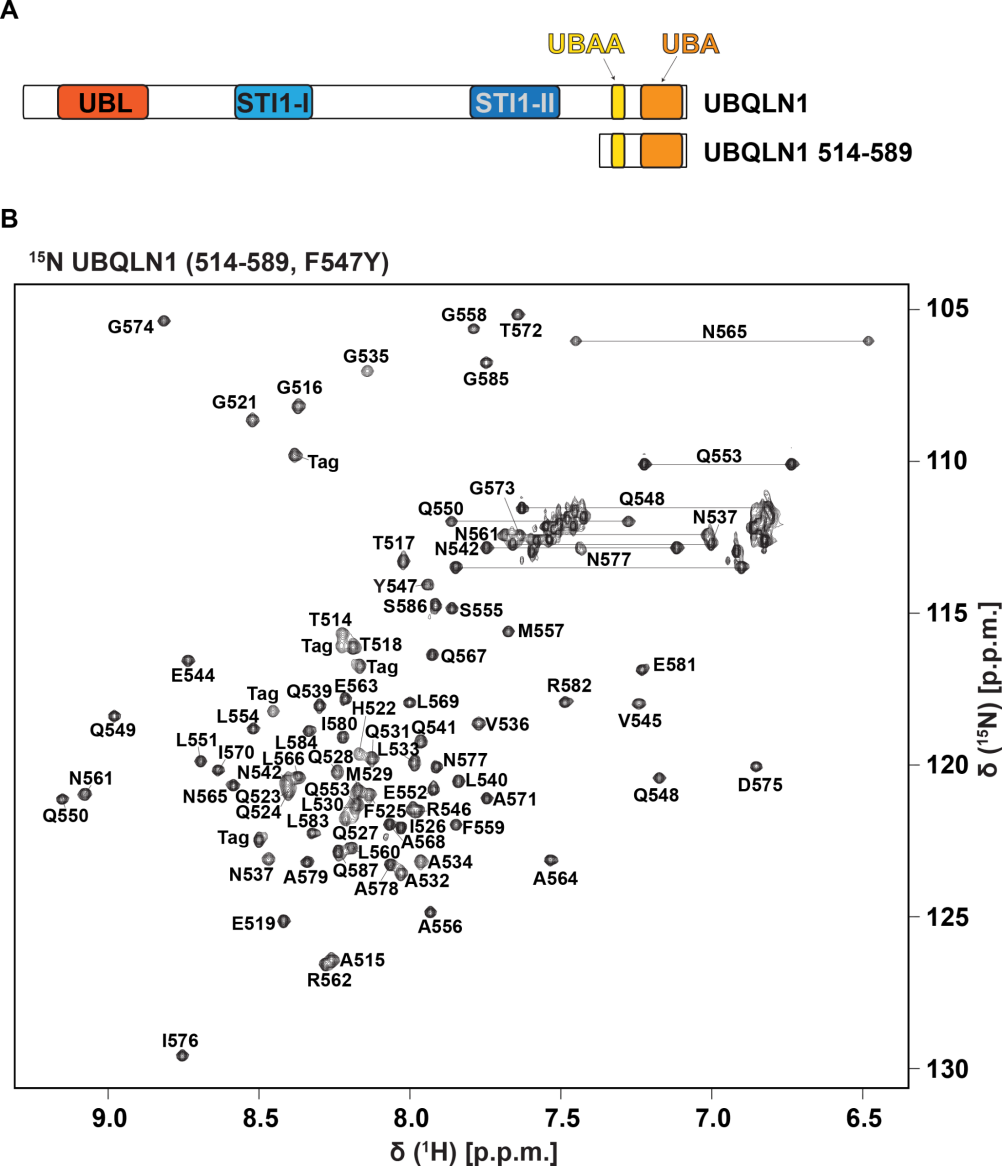
UBQLN1 domain layout and ^1^H-^15^N HSQC spectrum (**A**) Domain organization of UBQLN1. Full length UBQLN1 (top) and the protein fragment of the current study corresponding to residues 514–589 (bottom). (**B**) 2D ^1^H-^15^N HSQC spectrum of ^15^N-labeled UBQLN1 (514–589, F547Y) at 600 µM and 25 °C acquired on a Bruker Avance III 850 MHz spectrometer. Residue type and sequence position are included for peaks corresponding to backbone amides. Sidechain NH_2_ groups of asparagine and glutamine are also labeled

The UBQLNs (UBQLN1-4) are a family of proteasomal shuttle factors, so-named for their ability to simultaneously bind proteasomes and their ubiquitinated substrate proteins. They transiently bind to substrate receptors in the proteasome regulatory particle through their ubiquitin-like domain (UBL) (Chen et al. 2019; Walters et al. 2002) and interact with ubiquitin through their ubiquitin-associated domain (UBA) (Zhang et al. 2008). In addition to shuttling substrates to proteasomes, UBQLNs function as chaperones to prevent protein aggregation independently (Itakura et al. 2016), and in complex with other chaperones (Hjerpe et al. 2016). Well-ordered structures have been solved for the UBL and UBA (Walters et al. 2002; Zhang et al. 2008), which flank the two ends of the protein; however, much less is known about the central portion of the UBQLNs, which is predicted to be largely intrinsically disordered and to mediate multivalent interactions that contribute to biomolecular condensate formation (Dao et al. 2018). Here, we report the backbone (^N^H, N, C’, Cα, and Hα) and sidechain Cβ chemical shift assignments for the UBQLN1 region spanning 514–586 and amide (^N^H and N) resonance assignments for UBQLN1 spanning 514–589 with F547 replaced with tyrosine. These segments encompass the UBA as well as a region N-terminally adjacent to it called the UBA adjacent region (UBAA) (Fig. 1A). We find the chemical shift assignments in the region of the UBAA to be concentration-dependent, which is likely due to self-association mediated by the UBAA. These assignments may aid future NMR studies of UBQLN1 to understand the mechanism of UBAA self-association or other binding mechanisms in this region.

## Methods and experiments

### Expression and purification

**Fig. 2:**
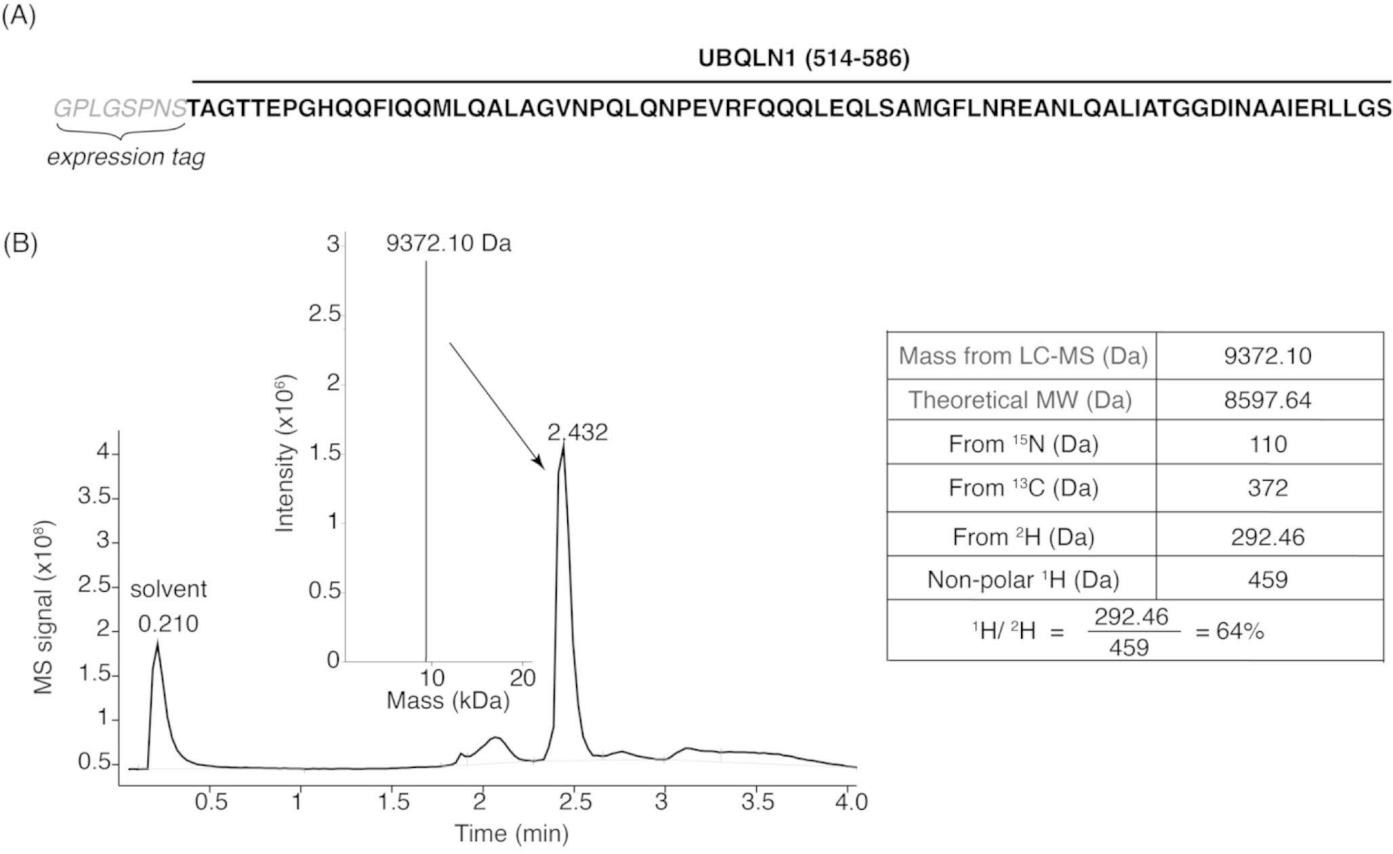
LC-MS analysis of purified ^15^N, ^13^C, ^2^H-labeled UBQLN1 (514–586) protein. (**A**) Amino acid sequence of UBQLN1 (514–586) and its N-terminal expression tag. (**B**) LC-MS analysis of purified ^15^N, ^13^C, ^2^H-labeled UBQLN1 (514–586) to estimate extent of deuterium labeling

UBQLN1 (514–586) and UBQLN1 (514–589, F547Y) were synthesized with codon optimization for expression in *E. coli* and inserted into the pGEX-6P-3 vector between the EcoRI and XhoI sites in frame with the PreScission protease cleavage site (GenScript). Plasmids were transformed into *E. coli* strain BL21(DE3) (Invitrogen C600003) in the presence of 100 µg/mL ampicillin. Transformants were grown in 10 mL of Luria-Bertani Broth (LB) overnight at 37 °C with shaking and centrifuged for 10 min at 2000 g. Bacterial pellets containing UBQLN1 constructs were gently resuspended into 1 L of M9 minimal media containing 1 g/L ^15^N ammonium chloride (Cambridge Isotope Laboratories NLM-467-1) as their only nitrogen source. Media used for UBQLN1 (514–586) additionally contained 2 g/L ^13^C glucose (Cambridge Isotope Laboratories CLM-1396) as the sole carbon source and was grown in 80% ^2^H_2_O/20% ^1^H_2_O (^2^H_2_O is from Cambridge Isotope Laboratories DLM-4-99-1000). Cells were grown at 37 °C with shaking until they reached an OD_600_ of 0.5–0.6 at which point IPTG was added to a concentration of 0.4 mM to induce protein expression at 17 °C overnight. Bacteria were pelleted at 4,000 rpm for 40 min at 4 °C in a Beckman Coulter J6-M1 centrifuge fitted with a JS-4.2 rotor and frozen at -80 °C until purification. Cell pellets were resuspended in lysis buffer (20 mM at Tris pH 7.6, 300 mM NaCl, 5 mM DTT, and Roche Complete Mini protease inhibitor cocktail (Roche Diagnostics 11836153001)). Resuspended bacteria were sonicated and centrifuged at 15,000 rpm (~ 27,216 g) at 4 °C for 30 min. Supernatants were incubated with glutathione Sepharose beads (Cytiva 17-0756-05) with fresh protease cocktail inhibitor added, for 3 h at 4 °C with agitation. Beads were washed 4–5 times in lysis buffer without protease inhibitors and incubated overnight with PreScission protease (Cytiva 27084301) at 4 °C with agitation to release UBQLN protein from the GST tag. UBQLN1 proteins were eluted from the beads in lysis buffer and then purified further by size exclusion chromatography on an ÄKTA pure FPLC system (Cytiva) using a HiLoad 16/600 Superdex75 prep grade column in FPLC buffer (10 mM MOPS at pH 6.5, 50 mM NaCl, 5 mM DTT, and 10 µM zinc sulfate). UBQLN1 proteins were concentrated with Amicon Ultra-15 filters with a 3 kDa cutoff (EMD Millipore UFC900324). Liquid chromatography-mass spectrometry (LC-MS) was performed with protein samples (10 µM) with 10% acetonitrile on a 6100 Series Quadrupole LC-MS (Agilent Technologies, Inc.), equipped with an electrospray source and operated in the positive ion mode. Data acquisition and analyses were performed using OpenLAB CDS ChemStation Edition C.01.05 (Agilent Technologies, Inc.). Based on the Mass (9370.20 Da) from LC-MS (Fig. 2), deuterium labeling of UBQLN1 (514–586) was estimated to be ~ 64%.

### NMR experiments

All NMR experiments were conducted in NMR buffer (10 mM MOPS at pH 6.5, 50 mM NaCl, 5 mM DTT, 10 µM zinc sulfate, 1 mM pefabloc, 0.1% NaN_3_, and 5% ^2^H_2_O/95% ^1^H_2_O). Experiments were performed with 0.35 mM ^15^N, ^13^C, ~ 64% ^2^H-labeled UBQLN1 (514–586), or ^15^N-labeled UBQLN1 (514–589, F547Y) at 50 µM or 600 µM. All NMR experiments were performed at 25 °C on Bruker Avance III 600, 800, or 850 MHz spectrometers equipped with cryogenically cooled probes. Spectra recorded for backbone assignments of UBQLN1 514–586 were 2D ^1^H-^15^N HSQC and ^1^H-^13^C HSQC, and 3D HNCACB, CBCA(CO)NH, HNCO, HN(CA)CO, and ^15^N-dispersed NOESY (200 ms mixing time). Spectra recorded for UBQLN1 514–589, F547Y were 2D ^1^H-^15^N HSQC and 3D ^15^N-dispersed NOESY (120 ms mixing time). All NMR data processing was performed using NMRpipe (Delaglio et al. 1995) and spectra were analyzed in XEASY (Bartels et al. 1995).

### Extent of assignments and data deposition

Assignments were completed for all backbone atoms of UBQLN1 514–586. Excluding residual non-native residues left behind from removal of the GST tag, 100% of backbone N, HN, C’, Cα, and Cβ atoms were assigned as well as 96% of Hα atoms (missing T514, H522, and P543). The ^15^N-dispersed NOESY experiment was used to validate Hα assignments. The F547Y substitution was introduced into the UBQLN1 514–589 construct to enable protein quantitation at 280 nm absorbance, and assignment of UBQLN1 514–589, F547Y was guided by assignments made for UBQLN1 514–586 (Fig. 1B). Of note, the nitrogen of the T572 backbone amide displays a chemical shift value of 105.2 ppm, which is ~1.6 standard deviations upfield compared to the average value for a threonine amide nitrogen (115.4 p.p.m., according to the BMRB website, calculated from the BMRB database, https://bmrb.io/ref_info/csstats). In concurrent work (Buel et al. 2023), we find that Hγ1 of T572 undergoes hydrogen bonding, likely to surrounding backbone carbonyls, and we expect that the upfield shifting of the T572 amide nitrogen is reflective of higher electron shielding afforded by the T572 Hγ1 hydrogen bond.

**Fig. 3:**
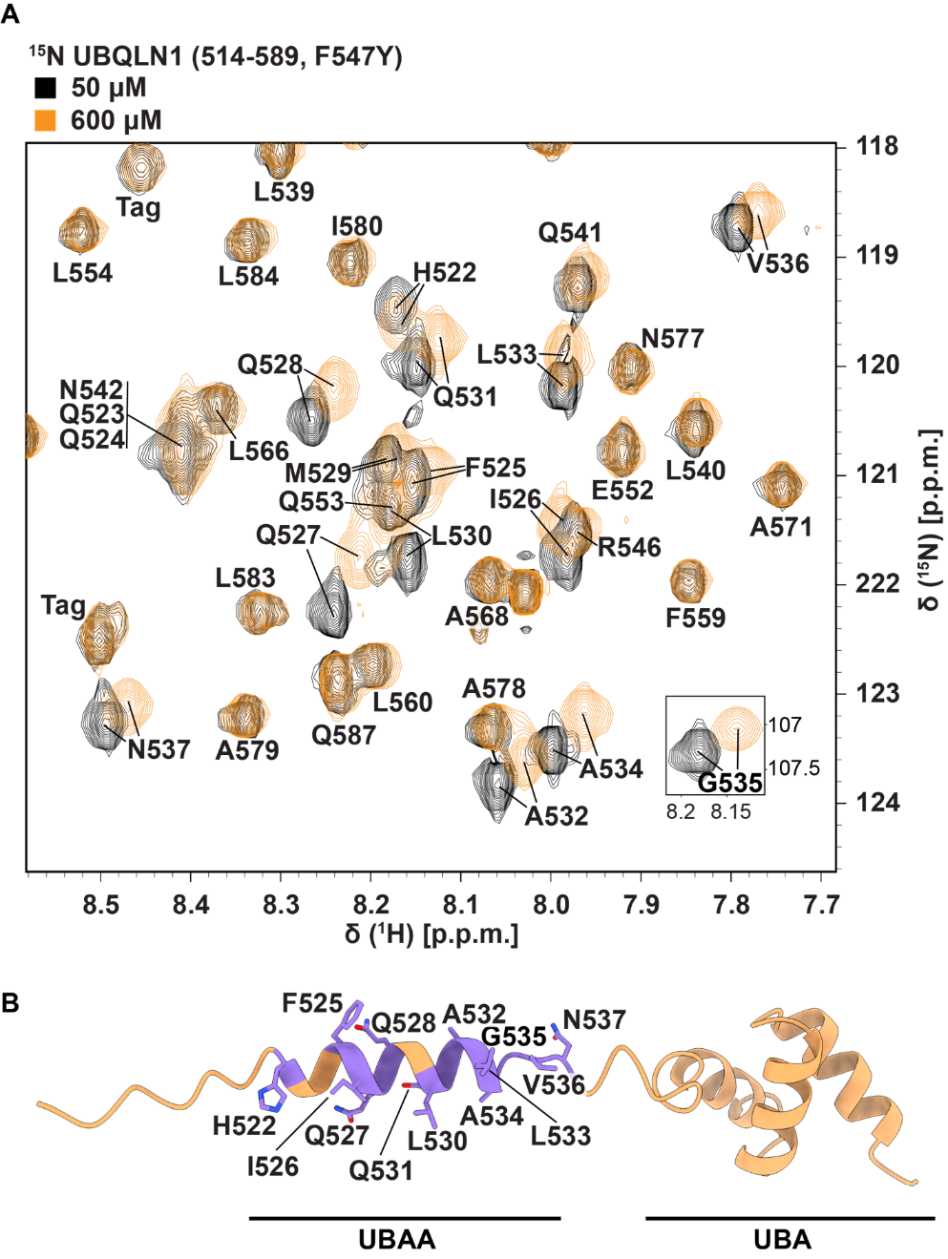
**UBQLN1 concentration-dependent assignments.** (**A**) Overlay of an expanded region of ^1^H-^15^N-HSQC spectra recorded on ^15^N-UBQLN1 (514–589, F547Y) at 50 µM (black) and 600 µM (orange) protein concentration to highlight differences in amide chemical shift values. These spectra were acquired at 25 °C on a Bruker Avance III 850 MHz spectrometer. (**B**) The residues with concentration-dependent chemical shift perturbations (CSPs) above 0.05 parts per million are mapped onto the AlphaFold2-predicted structure for UBQLN1 514–589. CSPs were calculated as $CSP = \sqrt {0.2\Delta\delta _N^2 + \Delta\delta _H^2}$, where Δδ_H_ is the change in amide proton value (in parts per million) and Δδ_N_ is the change in amide nitrogen value (in parts per million)

While working with UBQLN1 514–589, F547Y, residues in the region of H522-N537 were found to have concentration-dependent chemical shifts (Fig. 3A,B). We think this is due to self-association of UBQLN1 514–589, F547Y monomers in solution. We found in concurrent studies that protein binding to the nearby UBA allosterically induces changes in the UBAA region as observed through chemical shift perturbation analyses (Buel et al. 2023), and we therefore recommend exercising caution when interpreting chemical shift perturbation data in this region. Assignments for UBQLN1 514–586 have been deposited in the Biological Magnetic Resonance Data Bank (BMRB) under accession code 51768. Assignments for UBQLN1 514–589, F547Y were deposited to the BMRB at two concentrations: 50 µM (BMRB 51769) and 600 µM (BMRB 51770).

## Data Availability

Assignments for UBQLN1 514–586 have been deposited in the Biological Magnetic Resonance Data Bank (BMRB) under accession code 51768. Assignments for UBQLN1 514–589, F547Y were deposited to the BMRB at two concentrations: 50 µM (BMRB 51769) and 600 µM (BMRB 51770). Plasmids used for generation of proteins used in this study are available upon request.
